# A case of IgG4-related retroperitoneal fibrosis with significant involvement of the abdominal aorta—a clinical and diagnostic challenge

**DOI:** 10.1016/j.jvscit.2022.02.003

**Published:** 2022-03-08

**Authors:** Sinead Gormley, Paola Tacuri Bravo, Xavier Kos, Kamal Solanki, Manar Khashram

**Affiliations:** a Department of Vascular and Endovascular Surgery, Waikato Hospital, Hamilton, New Zealand; b Department of Histology, Waikato Hospital, Hamilton, New Zealand; c Department of Interventional Radiology, Waikato Hospital, Hamilton, New Zealand; d Department of Rheumatology, Waikato Hospital, Hamilton, New Zealand

**Keywords:** Abdominal aorta, Case report, IgG4-related disease, Pancreatitis, Retroperitoneal fibrosis

## Abstract

Immunoglobulin (Ig)G4-related disease (IgG4-RD) with retroperitoneal fibrosis (RPF) is a rare, fibroinflammatory disease involving the soft tissues of the retroperitoneum. A 73-year-old man with IgG4-related RPF affecting the abdominal aorta and iliac arteries was treated with steroids and mycophenolate mofetil. The prevalence of the disease remains unknown because it is often misdiagnosed and can mimic many malignant, infectious, and inflammatory conditions. Autoimmune pancreatitis is a common presenting condition of IgG4-RD. Because As IgG4-RD is responsive to steroids, diagnosing IgG4-related RPF early can prevent the exposure of patients with RPF to unnecessary diagnostic and therapeutic interventions.

Retroperitoneal fibrosis (RPF) is a rare heterogeneous disease of unknown etiology characterized by inflammation and fibrosis of the retroperitoneum that often presents a diagnostic challenge. Immunoglobulin (Ig)G4-related RPF (IgG4-RPF) is a progressive fibroinflammatory immune-mediated disease characterized by mass-forming, potentially destructive, inflammation and fibrosis in the soft tissues of the retroperitoneum. It is associated with elevation of serum IgG4 levels and infiltration of IgG4-positive plasma cells in single or multiple organ tissue sites. The diagnosis will often be delayed owing to an absence of clinical symptoms or because differentiation from other disease processes can be challenging. Therefore, a high level of diligence is required in the investigation of this condition when it is suspected. We have reported such a case of IgG4-related disease (IgG4-RD) with RPF affecting the abdominal aorta and common iliac arteries. The patient provided written informed consent for the report of his case details and imaging studies.

## Case report

**Fig 1:**
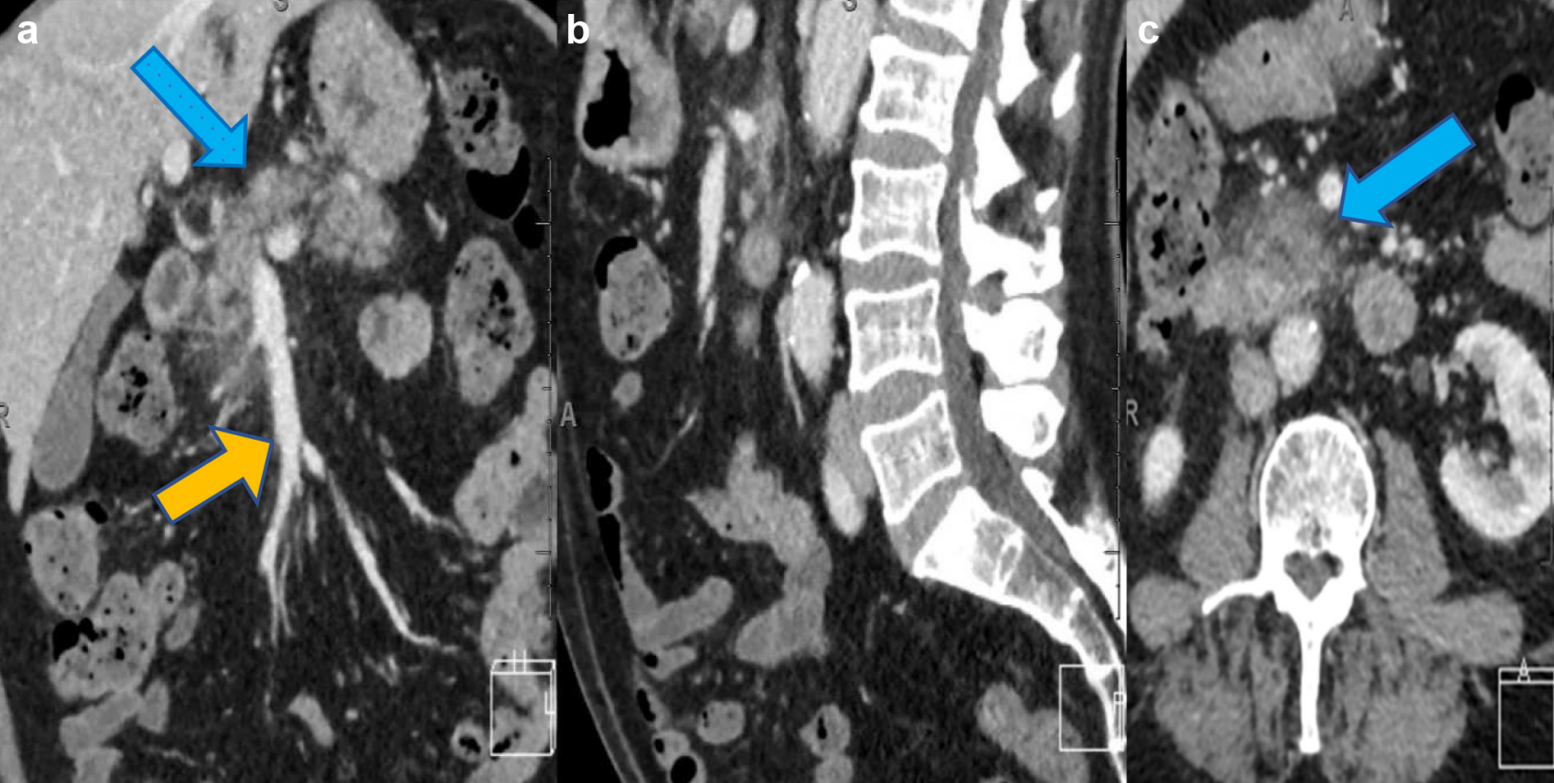
**a-c,** Computed tomography (CT) scans at the episode of pancreatitis 20 months before his presentation with immunoglobulin (Ig)G4-related retroperitoneal fibrosis (RPF) showing moderate peripancreatic fat stranding, particularly around the pancreatic head (*blue arrows*), with a normal-appearing aorta of normal caliber (*orange arrow*).

A 73-year-old white man had presented to the emergency department with a 3-week history of left flank pain radiating to the groin and associated with dark urine. His background included hypercholesterolemia, chronic obstructive pulmonary disease, and an episode of pancreatitis 20 months previously that had been suspected to be related to statin use (Fig 1). He denied any fevers, weight loss, or other urinary symptoms, including hematuria.

On physical examination, his height was 181 cm and his weight was 86 kg. His blood pressure was 177/89 mm Hg, and his heart rate was 65 bpm. His abdomen was soft, with some tenderness in the lower abdomen and both flanks. He had no skin or subcutaneous lesions. His full blood count and renal and liver function test results were normal, his erythrocyte sedimentation rate was 82 mm/h, and his C-reactive protein level was 36 mg/L. His serum IgG4 levels were increased at 3.26 g/L (reference range, 0.08-1.65 g/L), and the test results for rheumatoid factor, antithyroid peroxidase antibodies, antinuclear antibodies, antineutrophil cytoplasmic antibodies, and PR3/myeloperoxidase antibodies were negative. In addition, serology for hepatitis B and C virus, human immunodeficiency virus, syphilis, and *Mycobacterium tuberculosis* (QuantiFERON-TB Gold; Qiagen, Hilden, Germany) were negative.

**Fig 2:**
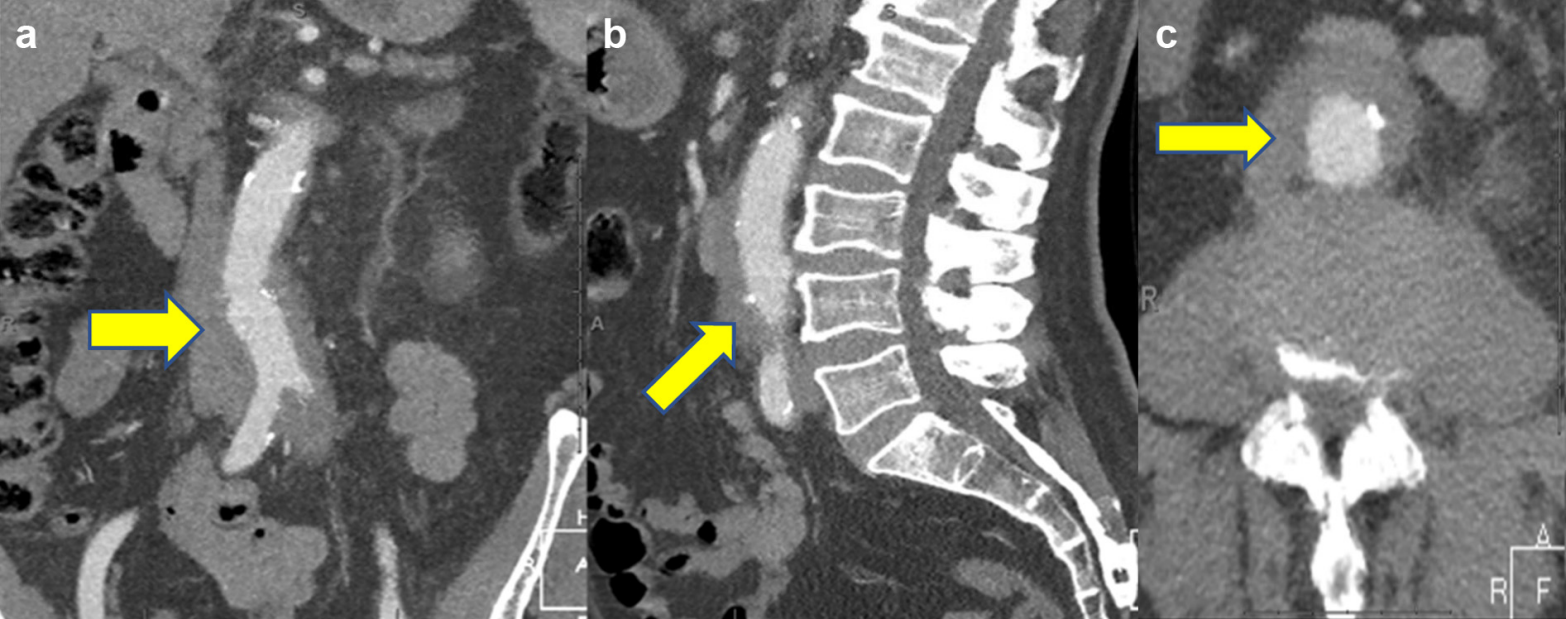
**a-c,** Computed tomography (CT) scans demonstrating extensive para-aortic soft tissue changes around the abdominal aorta and common iliac arteries (*yellow arrows*) causing left ureteral obstruction and moderate hydronephrosis of the left kidney, in keeping with retroperitoneal fibrosis (RPF). No associated abdominal aortic aneurysm was present.

On presentation, a computed tomography (CT) angiogram showed extensive para-aortic soft tissue changes around the abdominal aorta and common iliac arteries causing left ureteral obstruction and moderate hydronephrosis of the left kidney (Fig 2). At this stage, an inflammatory abdominal aortic aneurysm was strongly suspected, with a differential diagnosis of aortitis vs RPF. Also considered in the differential diagnosis were idiopathic RPF, IgG4-RD, malignancy, Rosai-Dorfman Destombes disease, and Erdheim-Chester disease.

**Fig 3:**
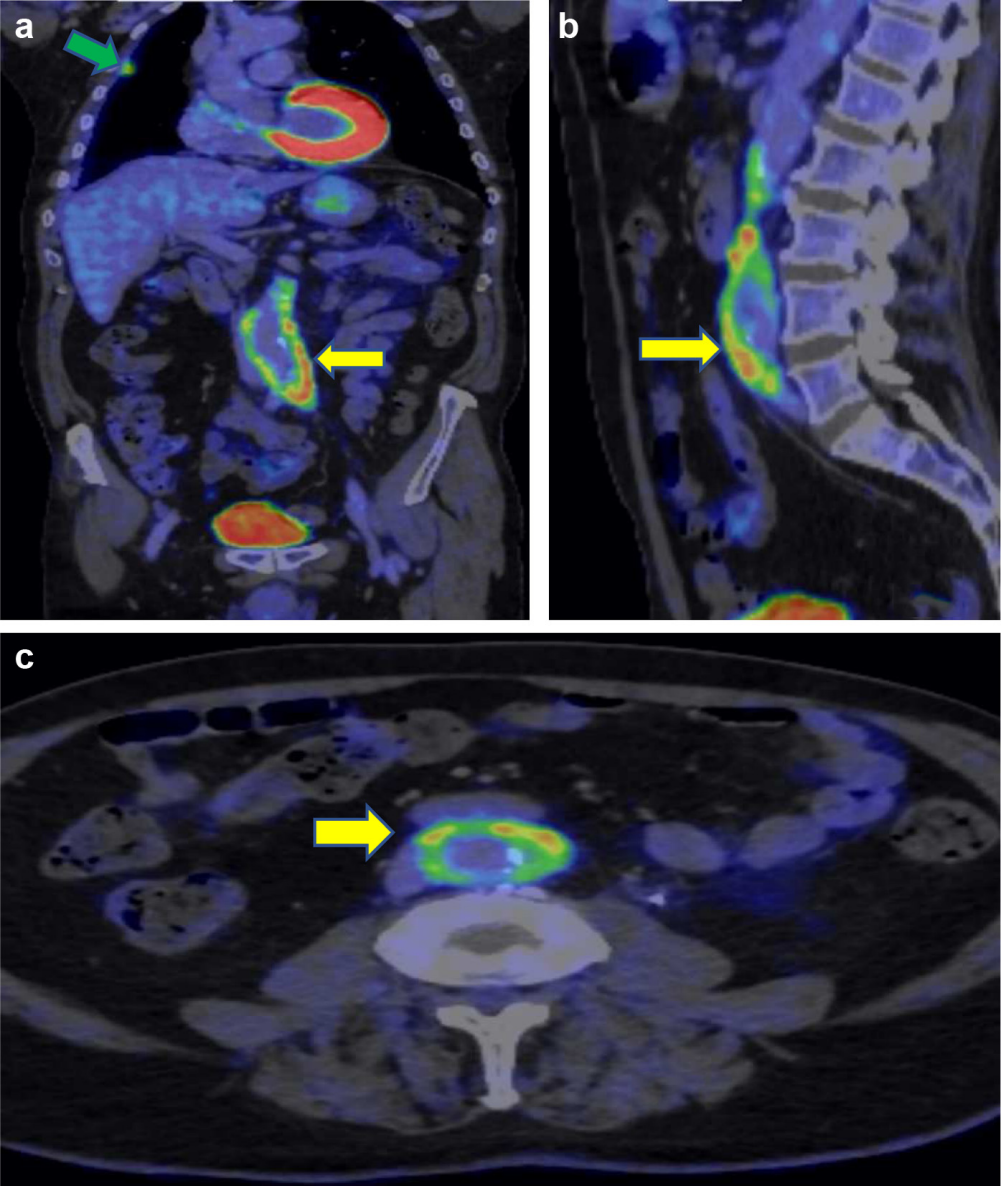
**a-c,** Positron emission tomography (PET)-computed tomography (CT) scans demonstrating extensive para-aortic soft tissue thickening with significant isotope uptake (*yellow arrows*), soft tissue thickening with increased uptake in the anterior mediastinum (*green arrow*).

**Fig 4:**
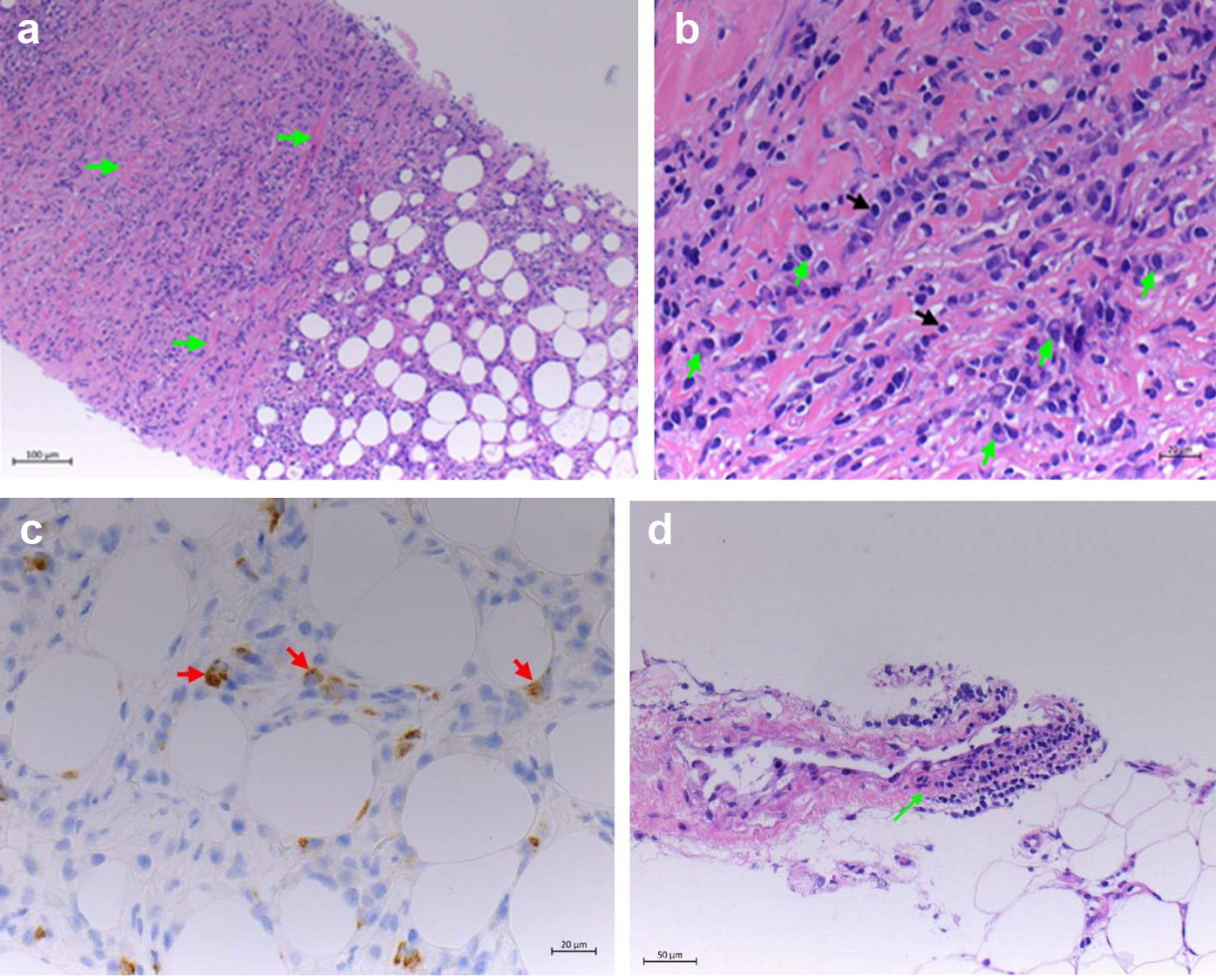
**a,** Histologic slide showing storiform fibrosis (*green arrows*; hematoxylin and eosin stain, original magnification ×10). **b,** Histologic slide showing plasma cells (*green arrows*; hematoxylin and eosin stain, original magnification ×40).

A subsequent positron emission tomography (PET)-CT scan (Fig 3) revealed para-aortic soft tissue thickening with significant isotope uptake thought to be inflammatory in nature. Increased avidity was also present in the anterior mediastinum, along with bilateral pleural thickening. A biopsy of the mediastinal tissue was performed, with histochemical examination confirming increased plasma cells, positivity for IgG4, and storiform fibrosis (Fig 4).

**Fig 5:**
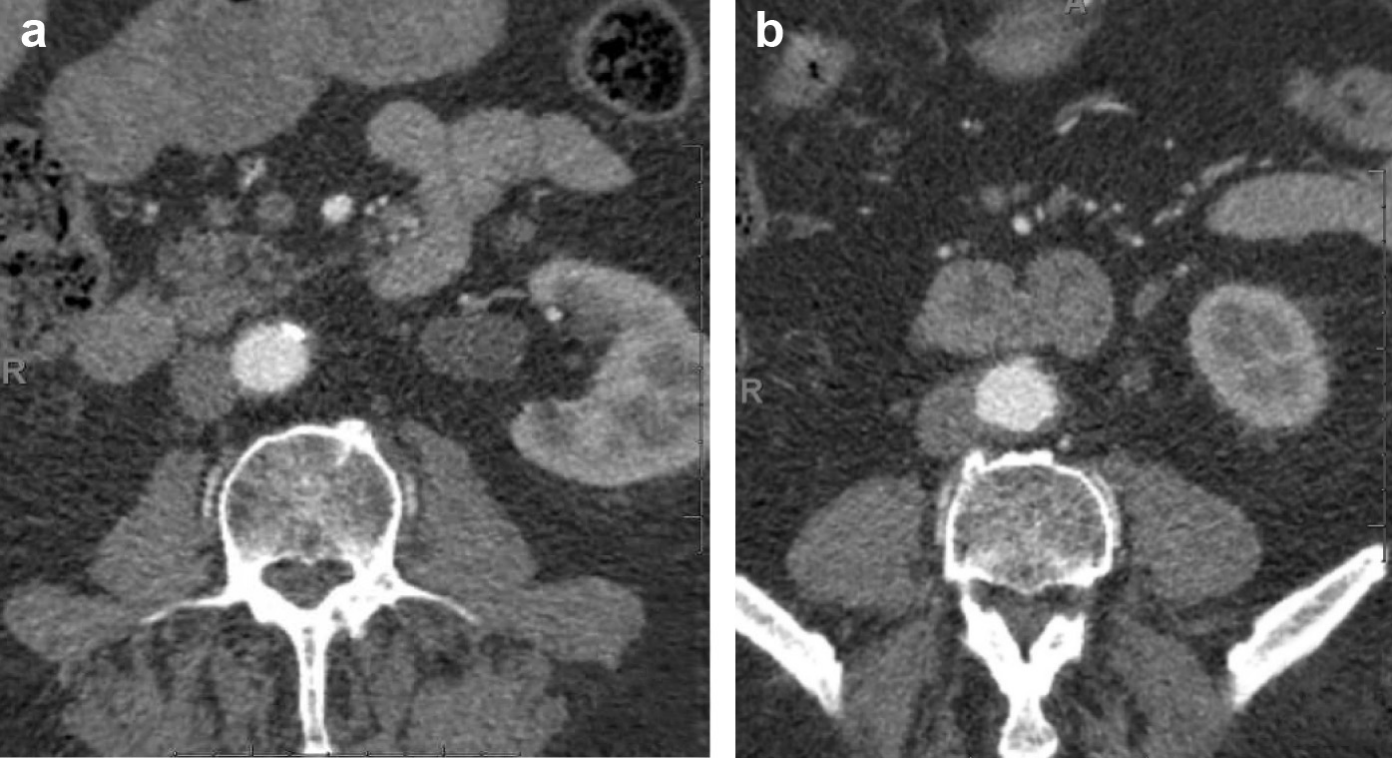
**a,b,** Computed tomography (CT) scans at 12 months demonstrating resolution of retroperitoneal soft tissue changes following treatment with corticosteroids and mycophenolate mofetil.

IgG4-RPF was diagnosed, and the patient began oral steroid therapy (1 mg/kg weight) and mycophenolate mofetil, which was titrated to 1 g twice daily. A ureteral stent was inserted to relieve the hydronephrosis. A repeat CT angiogram at 1 month demonstrated a stable appearance of the aortic wall thickening with no relapse of the hydronephrosis. Repeat CT and magnetic resonance imaging scans of the chest, abdomen, and pelvis at 12 months after his initial presentation demonstrated resolution of the retroperitoneal soft tissue changes with no evidence of hydronephrosis or ureteral dilatation. The mediastinal disease noted on the PET-CT scan had also resolved (Fig 5). Clinically, he remained asymptomatic, with no signs of relapse at 15 months of follow-up.

## Discussion

RPF is a rare disorder characterized by the presence of chronic inflammation and fibrosis in the retroperitoneal space.^1^ RPF was first described by the French urologist Albarran in 1905 as ureteral obstruction secondary to fibrotic changes in the retroperitoneal space. In 1948, Ormond described two more cases, which established RPF as a clinical entity. Its incidence varies from 0.1 to 2 per 100,000 persons. RPF occurs predominately in men, with a mean age at presentation of 40 to 65 years. No evidence has been demonstrated of familial clustering, and no clear ethnic predisposition has been shown. Two thirds of the cases are idiopathic, and other causes include malignancy, inflammatory periaortitis, autoimmune disease, retroperitoneal trauma, radiation, and certain medications.

IgG4-RD is a progressive fibroinflammatory condition believed to be secondary to dysregulation of the CD4 T-lymphocytes and plasmablasts. The hallmark features of IgG4-RD include lymphoplasmacytic inflammation, infiltration by IgG4-positive plasma cells, and distinct storiform fibrosis. It is a rare systemic autoimmune disease that can affect a variety of structures (eg, pancreas, biliary tract, salivary and lacrimal glands, lymph nodes, retroperitoneum). The correlation of autoimmune pancreatitis and elevated serum IgG4 levels was first identified in 2001 by Hamano et al,^2^ with IgG4-RD most often described in the pancreas. The multisystemic nature of IgG4-RD was recognized in 2003 when seven patients with an initial diagnosis of autoimmune pancreatitis were subsequently found to have extensive organ involvement with IgG4-positive plasma infiltration. The clinical manifestations of systemic IgG4-RD include type 1 autoimmune pancreatitis, RPF, cholangitis, and sialadenitis. For our patient, it was questioned whether his previous episode of pancreatitis was IgG4 related; however, the IgG4 levels had not been evaluated during that admission and, thus, IgG4-related pancreatitis could not be confirmed.

The characteristics of IgG4-RPF are similar to those of idiopathic RPF, except that a male predominance, older age, and greater incidence of postrenal acute kidney injury are prominent with IgG4-RPF.^3^ RPF usually involves the adventitia of the abdominal aorta, iliac arteries, and adjacent structures, resulting in flank pain, lower extremity edema, ureteral obstruction, and hydronephrosis. Early symptoms include abdominal or lumbar back pain and lower extremity swelling. Late symptoms include hypertension, mesenteric ischemia, bowel obstruction, acute kidney injury, anuria, uremia, and deep vein thrombosis. Also differing from other autoimmune diseases, IgG4-RPF is rarely accompanied by fever or joint pain.^4^ RPF has often been misdiagnosed as retroperitoneal visceral malignancy and treated with surgery.^4^ Therefore, early detection of IgG4-RD and treatment is important because of its good clinical response to glucocorticoids.

IgG4-RD often involves the aorta and includes IgG4 aortitis and IgG4 (chronic) periaortitis or periarteritis (IgG4-CP). Chronic periaortitis is a rare fibroinflammatory disorder that incorporates idiopathic RPF, inflammatory abdominal aortic aneurysms, perianeurysmal RPF, and IgG4-related periaortitis. Aortic involvement in IgG4-RD can be complicated by aortic wall dilatation, dissections, and inflammatory aneurysms. Aortitis must be treated urgently because inflammatory aneurysms can have a rapid growth rate and are at risk of rupture if they have a large diameter. Ig4-CP most predominately affects the infrarenal abdominal aorta and iliac arteries and can overlap with IgG4-RPF. It can be difficult to differentiate between IgG4-CP and IgG4-RPF, such as in our case, because both conditions can demonstrate similar radiologic findings of nonspecific inflammatory or fibrotic tissues in the periaortic retroperitoneum that encases adjacent structures. Similar to IgG4-RPF, patients with IgG4-CP will demonstrate a good clinical response to steroids. In general, no significant differences exist in the response to therapy and relapse rates between these two groups. However, theoretically, the risk of developing aortic aneurysms is increased with IgG4-CP, which should alert the physician to perform close monitoring for this complication.

The reference standard for the diagnosis of IgG4-RD involves confirmation of the histologic features such as storiform fibrosis, which is characteristic of IgG4-RPF and is more sensitive and specific than elevated serum IgG4 levels. Umehara et al^5^ proposed comprehensive diagnostic criteria in 2012 and highlighted that for a definite diagnosis, histologic examination is essential, especially for patients with atypical radiographic features. A study by Pucar and Hinchcliff^6^ presented the highest quality evidence regarding the potential value of dedicated ^18^F-fluorodeoxyglucose PET-CT examinations for the detection of vascular inflammation in patients with IgG4-RD. It can be difficult to obtain tissue samples from the retroperitoneal lesions; thus, ^18^F-fluorodeoxyglucose PET can be used to reveal sites of other active inflammatory lesions more amenable to tissue sampling, such as the mediastinal lesions in our patient. Otherwise, para-aortic tissue would need to be considered for biopsy. This can be a difficult and challenging area to access for biopsy and is a high-risk procedure owing to the potential arterial complications.

Khosroshahi et al^7^ reported an international consensus guidance statement on the treatment of IgG4-RD. Decisions on treatment strategies have been largely based on case reports and cohort groups. The treatment of IgG4-related RPF is challenging and mostly consists of long-term glucocorticoids.^8^ Given the heterogeneous nature of the disease and how it affects multiple organs with varying degree of aggressiveness, the treatment duration will vary, depending on which organs are involved and the degree of dysfunction. Assessing the efficacy of treatment involves close monitoring of the clinical features and serum IgG4 levels and inflammatory markers. Once these markers have decreased, the dose of the immunosuppressive agent should be slowly withdrawn, with continuous close monitoring.

Almeqdadi et al^9^ described the potential therapeutic role of rituximab in the treatment of IgG4-RD as an alternative to glucocorticoids. Corticosteroids tend to achieve prompt improvement in symptoms and often lead to a decrease in the size of the retroperitoneal lesions and resolution of obstructive complications. However, in the long term, corticosteroids can cause significant side effects and morbidity. Some patients will require intervention urgently because uncontrolled disease in certain major organs can result in irreversible damage. The outcome of idiopathic and benign forms of RPF will generally be good. However, malignant RPF carries a poor prognosis.^10^ “Malignant” IgG4-RPF refers to a severe aggressive type of IgG4-RPF resulting in organ dysfunction of a significant degree, leading to increased morbidity or, even, death, if not treated aggressively. Malignancy can be a cause of malignant IgG4-RPF. IgG4-RD has also been associated with an increased risk of malignancy, especially lymphoma and pancreatic cancer. Therefore, it is of great importance to consider screening patients for cancers in the management and diagnosis of IgG4-RD.

## Conclusions

RPF due to IgG4-RD is a rare clinical condition, and the spectrum of clinical manifestations is highly varied. Early diagnosis and differentiating IgG4-RPF from other diseases can result in success with medical treatment and prevent unnecessary surgical intervention. In the present case, we demonstrated successful resolution with the use of corticosteroids and immunomodulation with mycophenolate mofetil.

## References

[1] Tanaka T., Masumori N. (2020). Current approach to diagnosis and management of retroperitoneal fibrosis. Int J Urol.

[2] Hamano H., Kawa S., Horiuchi A., Unno H., Furuya N., Akamatsu T. (2001). High serum IgG4 concentrations in patients with sclerosing pancreatitis. N Engl J Med.

[3] Cronin C.G., Lohan D.G., Blake M.A., Roche C., McCarthy P., Murphy J.M. (2008). Retroperitoneal fibrosis: a review of clinical features and imaging findings. AJR Am J Roentgenol.

[4] Lian L., Wang C., Tian J.-L. (2016). IgG4-related retroperitoneal fibrosis: a newly characterized disease. Int J Rheum Dis.

[5] Umehara H., Okazaki K., Masaki Y., Kawano M., Yamamoto M., Saeki T. (2012). Comprehensive diagnostic criteria for IgG4-related disease (IgG4-RD), 2011. Mod Rheumatol.

[6] Pucar D., Hinchcliff M. (2021). FDG PET vascular imaging in IgG4-RD: potential and challenges. J Nucl Cardiol.

[7] Khosroshahi A., Wallace Z.S., Crowe J.L., Akamizu T., Azumi A., Carruthers M.N. (2015). International consensus guidance statement on the management and treatment of IgG4-related disease. Arthritis Rheumatol.

[8] Mota M.M.D.S., Bezerra R.O.F., Garcia M.R.T. (2018). Practical approach to primary retroperitoneal masses in adults. Radiol Bras.

[9] Almeqdadi M., Al-Dulaimi M., Perepletchikov A., Tomera K., Jaber B.L. (2018). Rituximab for retroperitoneal fibrosis due to IgG4-related disease: a case report and literature review. Clin Nephrol Case Stud.

[10] Caiafa R.O., Vinuesa A.S., Izquierdo R.S., Brufau B.P., Colella J.R.A., Molina C.N. (2013). Retroperitoneal fibrosis: role of imaging in diagnosis and follow-up. Radiographics.

